# Apolipoprotein CIII Overexpression-Induced Hypertriglyceridemia Increases Nonalcoholic Fatty Liver Disease in Association with Inflammation and Cell Death

**DOI:** 10.1155/2017/1838679

**Published:** 2017-01-10

**Authors:** Adriene A. Paiva, Helena F. Raposo, Amarylis C. B. A. Wanschel, Tarlliza R. Nardelli, Helena C. F. Oliveira

**Affiliations:** Department of Structural and Functional Biology, Biology Institute, State University of Campinas, Campinas, SP, Brazil

## Abstract

Nonalcoholic fatty liver disease (NAFLD) is the principal manifestation of liver disease in obesity and metabolic syndrome. By comparing hypertriglyceridemic transgenic mice expressing apolipoprotein (apo) CIII with control nontransgenic (NTg) littermates, we demonstrated that overexpression of apoCIII, independent of a high-fat diet (HFD), produces NAFLD-like features, including increased liver lipid content; decreased antioxidant power; increased expression of TNF*α*, TNF*α* receptor, cleaved caspase-1, and interleukin-1*β*; decreased expression of adiponectin receptor-2; and increased cell death. This phenotype is aggravated and additional NAFLD features are differentially induced in apoCIII mice fed a HFD. HFD induced glucose intolerance together with increased gluconeogenesis, indicating hepatic insulin resistance. Additionally, the HFD led to marked increases in plasma TNF*α* (8-fold) and IL-6 (60%) in apoCIII mice. Cell death signaling (Bax/Bcl2), effector (caspase-3), and apoptosis were augmented in apoCIII mice regardless of whether a HFD or a low-fat diet was provided. Fenofibrate treatment reversed several of the effects associated with diet and apoCIII expression but did not normalize inflammatory traits even when liver lipid content was fully corrected. These results indicate that apoCIII and/or hypertriglyceridemia plays a major role in liver inflammation and cell death, which in turn increases susceptibility to and the severity of diet-induced NAFLD.

## 1. Introduction

Hypertriglyceridemia is a common condition caused by multiple environmental and genetic factors [1, 2]. Elevated plasma levels of triglyceride- (TG-) rich remnant lipoproteins are independent risk factors for cardiovascular disease (CVD) [3]. Clinical and experimental studies have shown strong correlations and causal links between plasma TG and apolipoprotein CIII (apoCIII) levels [4, 5]. Plasma apoCIII levels are also increased in individuals with diabetes [6, 7]. Moreover, loss-of-function mutations in the apoCIII gene are associated with low TG levels and a reduced risk of CVD [8, 9]. Therefore, TG levels are causally linked to apoCIII and CVD, and apoCIII inhibitors are already in clinical development to reduce CVD risk [10].

Hypertriglyceridemia and nonalcoholic fatty liver disease (NAFLD) are common features in obesity and metabolic syndrome [11]. The prevalence of NAFLD in western countries ranges from 25 to 35% [12], and liver steatosis is observed in 80% of individuals with obesity [13]. Hepatic insulin resistance and type II diabetes are considered sequelae of NAFLD [14]. Furthermore, persistent steatosis may progress to steatohepatitis (NASH), cirrhosis, and hepatocarcinoma [15].

The two-hit hypothesis [16] has been proposed to explain NAFLD pathogenesis. In this hypothesis, steatosis represents the “first hit.” Steatosis increases the vulnerability of the liver to various “second hits” that in turn lead to inflammation, fibrosis and cellular death. Oxidative stress is one such second hit. The inflammatory response, including the production of numerous proinflammatory molecules and adipokines, also has a key role in the initiation and progression of the disease [17]. Proinflammatory cytokines can cause liver damage either directly or indirectly by increasing oxidative stress; in turn, oxidative stress can impair liver function either directly or indirectly by perpetuating a vicious cycle [18]. The pathways that control oxidative stress and inflammation underlie many cardiometabolic diseases, including obesity, diabetes, and atherosclerosis. Accordingly, recent evidence suggests that the morbidity and mortality associated with NAFLD are not restricted to changes in the liver, as the majority of deaths of patients with NAFLD are related to CVD [19].

We previously demonstrated that hypertriglyceridemic transgenic mice overexpressing apoCIII exhibit increases in hepatic glycerolipid content and liver oxidative stress. The latter was associated with increased NADPH oxidase and xanthine oxidase activities, even when the mice consumed a regular low-fat diet (LFD) [20]. Another recent study reported that apoCIII-overexpressing mice develop NAFLD associated with severe hepatic insulin resistance, increased liver lipid uptake and decreased lipid secretion following consumption of a high-fat diet (HFD) [21].

The present study was designed to investigate whether apoCIII overexpression and/or the resulting hypertriglyceridemia trigger the main events driving the evolution of steatosis to NASH, namely, inflammation and cell death. Furthermore, we tested whether the PPARa agonist fenofibrate, which regulates many genes related to inflammation and lipid metabolism, including apoCIII, could reduce susceptibility to NAFLD.

## 2. Materials and Methods

### 2.1. Animals and Treatments

All experimental protocols for this study were approved by the university's Committee for Ethics in Animal Experimentation (CEUA/UNICAMP, protocol number 2436), and the research was conducted in conformity with the Public Health Service Policy. Male mice transgenic for human apoCIII and nontransgenic controls were maintained at the Division of Physiology and Biophysics, Biology Institute, State University of Campinas (São Paulo, Brazil). Human apoCIII transgenic founder mice (line 3707) [22] were donated by Dr. Alan R. Tall (Columbia University, New York, NY) in 1996 and have since been crossbred with wild-type (NTg) C57BL/6 mice (Multidisciplinary Center for Biological Research of the University of Campinas). The apoCIII transgenic mice were screened according to their fasting TG plasma levels (apoCIII mice > 300 mg/dL; control mice < 100 mg/dL) and housed in a room at 22°C ± 2°C with a 12-hour light-dark cycle with free access to water and food. One-month-old male mice (transgenic and NTg littermates) were fed either a LFD or a HFD until 4 months of age. Additional groups of mice fed a HFD were treated with fenofibrate (100 mg/kg bw, Allergan, SP, Brazil, solubilized in 5% Arabic gum) or 5% Arabic gum (control untreated group) during the last 2 weeks of HFD consumption by daily gavage. At 4 months of age, fasted mice were anesthetized via intraperitoneal (IP) injection of ketamine and xylazine (50 and 10 mg/kg) and euthanized by exsanguination through the retro-orbital plexus (see Table 1).

### 2.2. Biochemical Analyses

Plasma levels of total cholesterol, triglycerides (Chod-Pap; Roche Diagnostic GmbH, Mannheim, Germany), nonesterified fatty acids (Wako Chemical, Neuss, Germany), and liver transaminases (Biotecnica, SP, Brazil) were assayed using enzymatic-colorimetric methods according to the manufacturers' instructions. Leptin, adiponectin (Merck Millipore, Darmstadt, Germany), C-reactive protein (IBL-America, Minneapolis, USA), TNF*α*, and IL-1*β* (R&D Systems, Minneapolis, USA) plasma concentrations were determined using ELISA. For analysis of IL-1*β* expression in liver tissue, 50-mg tissue samples were homogenized in 2 mL Lysis Buffer 2 (R&D Systems, Minneapolis, USA). IL-6 plasma levels were analyzed using a Multiplex Assay (Merck Millipore, Darmstadt, Germany). Protein carbonyl content in the liver was measured using a colorimetric assay kit (Cayman Chemical Company, Michigan, USA).

### 2.3. Levels of Reduced and Oxidized Glutathione in Liver and Plasma

Liver (50 mg) and plasma (50 *μ*L) levels of reduced glutathione (GSH) and oxidized glutathione (GSSG) were assayed separately according to the fluorometric orthophthalaldehyde (OPT) method reported by Hissin and Hilf [23]. This method is based on the fact that OPT reacts with GSH and GSSG at pH 8.0 and pH 12, respectively, to yield a highly fluorescent product that can be activated at 350 nm with an emission peak of 420 nm. GSSG levels were determined after sample treatment with N-ethylmaleimide to ensure complete removal of GSH. The concentrations of GSH and GSSG in samples were calculated according to standard curves individually prepared with GSH and GSSG.

### 2.4. Liver Triglyceride Content

Liver lipids were extracted using the Folch [24] method. The lipid extracts were resuspended in Triton buffer (20 mL of 0.5 M potassium phosphate, pH 7.4, 0.25 M NaCl, 25 mM cholic acid, 0.5% Triton® X-100), and TG levels were determined using an enzymatic-colorimetric method according to the manufacturer's instructions (Chod-Pap; Roche Diagnostic GmbH, Mannheim, Germany).

### 2.5. Liver Histological Analysis

Liver tissue samples were taken from the left lobe and incubated in 10% phosphate-buffered formaldehyde at room temperature overnight. The samples were then washed 3 times with phosphate-buffered saline (PBS) and fixed in 70% ethanol. After fixation, the tissues were embedded in paraffin, sectioned to a thickness of 5 *μ*m and stained with hematoxylin-eosin (HE).

### 2.6. Oil Red O Staining

Liver samples were fixed in 4% formaldehyde, washed with PBS, embedded in Tissue-Tek OCT embedding compound, and frozen. The frozen sections (10 *μ*m thick) were rehydrated, and neutral lipid accumulation was detected by Oil Red O staining. Then, the sections were rinsed with 60% isopropanol and stained for 18 min with prepared Oil Red O solution (0.5% in isopropanol followed by dilution to 60% with distilled water and filtration). Slides were washed twice in 60% isopropanol and distilled water. Digital images were taken with an Olympus BX51 microscope connected to an Olympus DP72 digital camera.

### 2.7. Immunofluorescence Microscopy

Liver sections were fixed in 4% paraformaldehyde in PBS for 30 min at room temperature and then washed with PBS. Nonspecific binding was blocked by incubation with 5% bovine serum albumin (BSA) in PBS for 1 h. The sections were then incubated with primary antibodies specific for TNFR1 (1 : 50, Santa Cruz Biotechnology) or IL1*β* (1 : 100, Cell Signaling) overnight (4°C), followed by incubation with a primary antibody against CD68 (1 : 250, Serotec) for 3 h (room temperature). Next, the tissue sections were incubated with Alexa Fluor-labeled secondary antibodies (Invitrogen) for 1 h (room temperature). Pictures were taken on a Leica DMI600B microscope, and colocalized areas in random fields (1 per section/mouse) were analyzed using ImageJ software.

### 2.8. Analysis of Apoptosis

Apoptosis was analyzed via the terminal deoxynucleotidyl transferase dUTP nick end labeling (TUNEL) method using an in situ cell detection kit (Roche Diagnostics). Nuclei were labeled with 4′,6-diamidino-2-phenylindole (DAPI; Sigma Aldrich). Only TUNEL-positive cells that colocalized with DAPI-stained nuclei were considered apoptotic and counted. Random fields (5 per section) of 10 *μ*m were counted for each mouse.

### 2.9. Oral Glucose Tolerance Test and Insulin Tolerance Test

For the oral glucose tolerance test (OGTT), after 12 hours of fasting, mice received an oral dose of glucose solution (1.5 g/kg body weight). Basal blood samples were collected from the tail tip before (*t* = 0 min) and 15, 30, 60, and 90 min after glucose ingestion. For the insulin tolerance test (ITT), mice were fasted for three 3 hours, and blood samples were collected immediately before IP insulin injection [0.75 U/Kg body weight of regular human insulin (Eli Lilly Co.)] and at 5, 10, 15, 30, and 60 min after injection for glucose analysis. Blood glucose concentrations were measured using a glucose analyzer (Accu-Chek Advantage, Roche Diagnostic, Switzerland).

### 2.10. Pyruvate-Derived Glucose Production Test

After 16 hours of fasting, mice were injected with a pyruvate solution (1.5 g/kg body weight). Blood samples were collected from the tail tip before (*t* = 0 min) and 15, 30, 60, and 90 min after the injection. Blood glucose concentrations were measured using a glucose analyzer (Accu-Chek Advantage, Roche Diagnostic, Switzerland).

### 2.11. Analysis of Liver Very Low-Density Lipoprotein-Triglyceride Secretion

After 12 h of fasting, basal blood samples were collected from mice via the tail tip (*t* = 0 min). Then, the mice received an IP injection of Triton WR 1339 (500 mg/kg in saline solution; Sigma) to inhibit lipoprotein lipase activity as well as TG hydrolysis and clearance. Additional blood samples were collected at 120 and 150 minutes after Triton injection. For analysis of very low-density lipoprotein- (VLDL-) TG secretion, plasma TG levels were determined using an enzymatic-colorimetric assay according to the manufacturer's instructions (Chod-Pap; Roche Diagnostic GmbH, Mannheim, Germany).

### 2.12. RNA Extraction and Real-Time Reverse Transcription PCR

Total liver RNA was extracted from approximately 50 mg of tissue using TRIzol reagent (Invitrogen, Grand Island, NY, USA). RNA integrity was assessed using Tris-borate 1.2% agarose gels stained with ethidium bromide. RNA quantity and purity were measured via optical density readings taken at 260 and 280 nm (Gene Quant, Amersham-Pharmacia Biotech). Genomic DNA contamination was excluded by running polymerase chain reaction (PCR) on the RNA samples. cDNA was prepared in duplicate from 2 *μ*g of total RNA via reverse transcription using an Applied Biosystems High-Capacity cDNA Reverse Transcription Kit according to the manufacturer's instructions. Gene expression was determined using real-time reverse transcription polymerase chain reaction (RT-PCR) (Step One Real-time PCR System, Applied Biosystems, Foster City, CA, USA) with SYBRGreen PCR Master Mix and specific primers. The ΔΔCT method was used to quantify gene expression. The threshold cycle was normalized to *β*-actin and then expressed relative to the control groups (see Table 2).

### 2.13. Western Blotting

Liver tissue samples were homogenized in urea lysis buffer (2 M thiourea, 5 mM EDTA, 1 mM sodium fluoride, 1 mM sodium orthovanadate, 1 mM sodium pyrophosphate, 1% aprotinin, 2 mM PMSF, and 1% Triton-X 100), and protein concentrations were determined using the Bradford [25] method. Forty-microgram samples of protein lysate were resolved on SDS-polyacrylamide gels, transferred to nitrocellulose membranes and stained with Ponceau S (Sigma) to verify transfer efficiency and equal sample loading. The membranes were blocked with 5% albumin in Tris-HCl pH 7.6 containing 150 mM sodium chloride and 0.1% Tween-20 (TBST) and incubated for 2 hours at room temperature with antibodies against caspase-1 (1 : 500, Abcam), caspase-3 (1 : 100, Santa Cruz Biotechnology), Bcl2 (1 : 1000, Cell Signaling), or Bax (1 : 1000, Cell Signaling). An antibody against tubulin was used as an internal control (1 : 20,000, Sigma). Then, the membranes were washed with TBST, incubated with horseradish peroxidase- (HRP-) conjugated secondary antibodies diluted 1 : 1000, and washed again. Reactions were developed using an enhanced chemiluminescence detection system (Pierce ECL Western Blotting Substrate, Thermo Scientific, Rockford, USA). Images were captured using ImageQuant LAS 400 Mini equipment, and band intensities were quantified via optical densitometry using ImageJ software.

### 2.14. Statistical Analysis

Results are presented as the mean ± standard error for the number of determinations (*n*) indicated. Statistical analysis was performed using two-way ANOVA followed by Bonferroni correction. Statistical significance was defined as *p* ≤ 0.05.

## 3. Results

### 3.1. ApoCIII Overexpression Increases Diet-Induced Adiposity

Hypertriglyceridemic apoCIII-overexpressing mice were compared to control nontransgenic (NTg) littermates following consumption of either a LFD or a HFD. Morphometric and plasma biochemical parameters were assessed (Table 3). The HFD increased daily caloric intake, body mass, and white perigonadal adipose tissue mass but decreased relative liver mass in both groups. The apoCIII mice fed the HFD showed greater adiposity accompanied by increased leptin plasma levels. As expected, there was a genotype-dependent but diet-independent hyperlipidemic phenotype in the apoCIII mice, as shown by elevated plasma levels of TG, cholesterol and free fatty acids (Table 3).

Next, we assessed several indicators related to the natural history of NAFLD, including lipid accumulation, redox imbalance, inflammation, and cell death.

### 3.2. Overexpression of ApoCIII Promotes Hepatic Steatosis and Liver Dysfunction

**Figure 1:**
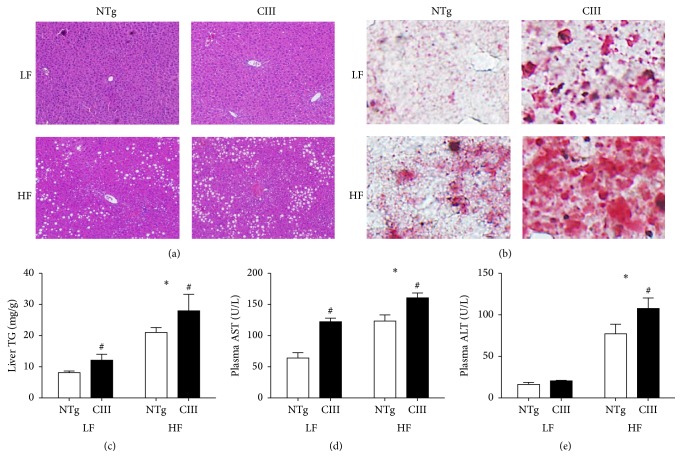
Overexpression of apoCIII promotes hepatic steatosis and dysfunction. NTg and apoCIII mice fed with low-fat (LF) or high-fat (HF) diets for 16 weeks. Representative liver sections isolated from mice stained with (a) HE and (b) Oil Red O. Note the macrovesicular lipid deposits, which appear as white spots in the HE-stained tissues (a), and the presence of lipids, which are stained in red (b). (c) Liver triglyceride content determined by enzymatic assay (*n* = 7-8). (d) Plasma concentrations of the transaminases ALT and AST (*n* = 5). Data are expressed as the mean ± SEM. ^*∗*^LF versus HF groups; ^#^NTg versus apoCIII mice (*p* < 0.05; two-way ANOVA).

Overexpression of apoCIII resulted in increased liver lipid content independent of diet type. This was observed as macrovesicular steatosis in histological analyses performed using HE (Figure 1(a)) and Oil Red O staining (Figure 1(b)) and as increased liver TG content (38% in the LFD group and 28% in the HFD group) (Figure 1(c)). Confirming the presence of liver injury, the apoCIII mice showed increased plasma levels of the hepatic transaminase AST under both diets and of ALT under the HFD (Figures 1(d) and 1(e)).

### 3.3. Overexpression of ApoCIII Promotes Glucose Intolerance and Increases Hepatic Glucose Production following Consumption of a HFD

**Figure 2:**
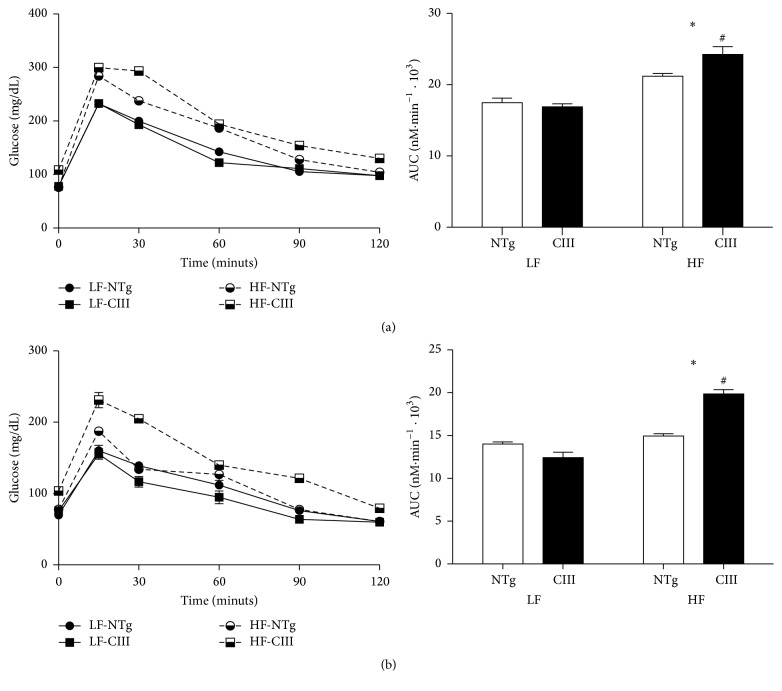
Overexpression of apoCIII promotes glucose intolerance and increases glucose hepatic production in mice consuming a high-fat diet. NTg and apoCIII mice were fed either a low-fat (LF) or high-fat (HF) diets for 16 weeks. (a) Glucose tolerance test results and area under the curve (AUC) (*n* = 7–9). (b) Pyruvate-derived glucose production and area under the curve (AUC) (*n* = 6–8). Data are expressed as the mean ± SEM. ^*∗*^LF versus HF groups; ^#^NTg versus apoCIII mice (*p* < 0.05; two-way ANOVA).

Disturbed glucose metabolism is associated with liver steatosis; therefore, we next evaluated glucose homeostasis in the mice. As shown in Figure 2, HFD consumption induced glucose intolerance in both groups, although the effect of the HFD was more potent in the apoCIII mice (Figure 2(a)), which showed a 12% increase in the area under the glycemic curve compared to the NTg group. The ITT results showed no diet or genotype effects (data not shown). However, in determining the pyruvate-derived liver glucose production rate (Figure 2(b)), we showed that the HFD-fed apoCIII mice had increased gluconeogenesis capacity. Together with the glucose intolerance exhibited by these mice, these results indicate that hepatic insulin resistance was present in the apoCIII mice fed the HFD.

### 3.4. Effects of ApoCIII Overexpression and HFD Consumption on Intracellular Lipid Metabolism-Related Gene Expression and Liver VLDL-TG Secretion

**Figure 3:**
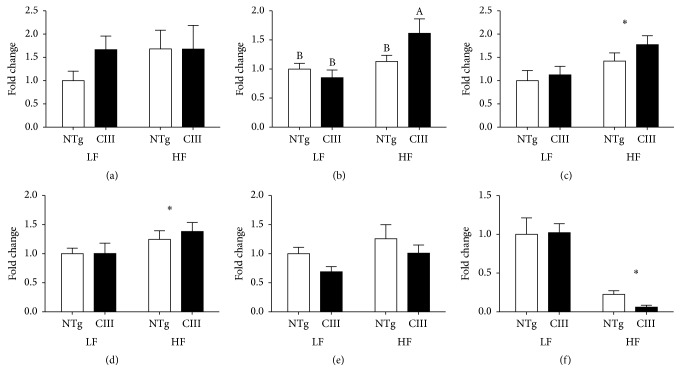
Effects of apoCIII overexpression and high-fat diet consumption on the expression of lipogenesis-related genes. mRNA expression levels for (a) CD36, (b) ChREBP, (c) SREBP1c, (d) ACC, (e) FAS, and (f) SCD1 in the livers of NTg and apoCIII mice fed either a low-fat (LF) or high-fat (HF) diets were analyzed and normalized to *β*-actin (*n* = 6–8). Data are expressed as the mean ± SEM. ^*∗*^LF versus HF group; ^#^NTg versus apoCIII mice (*p* < 0.05; two-way ANOVA). ^A, B^Mean values with nonmatching superscript letters are significantly different (*p* < 0.05; ANOVA followed by Bonferroni correction).

**Figure 4:**
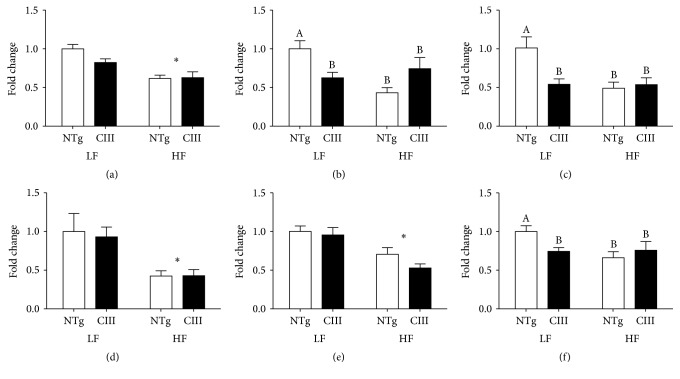
Effects of apoCIII overexpression and high-fat diet consumption on the expression of genes related to lipid catabolism and secretion. mRNA expression levels for (a) ATGL, (b) CPT1, (c) UCP2, (d) PGC1*α* (d), (e) MTP, and (f) APOB100 in the livers of NTg and apoCIII mice fed either a low-fat (LF) or high-fat (HF) diets and normalized to *β*-actin (*n* = 6–8). Data are expressed as the mean ± SEM. ^*∗*^LF versus HF group; ^#^NTg versus CIII mice (*p* < 0.05; two-way ANOVA). ^A, B^Mean values with nonmatching superscript letters are significantly different (*p* < 0.05; ANOVA followed by Bonferroni correction).

To identify the processes driving the observed increases in liver lipid content, we analyzed the expression of genes related to lipid uptake, synthesis, catabolism, and secretion (Figures 3 and 4). We observed that the mRNA expression of CD36, which is responsible for lipid uptake, was not significantly modulated by diet or genotype (Figure 3). In contrast, mRNA expression of ChREBP (carbohydrate response element binding protein), a transcription factor involved in lipogenesis and activated by glucose, increased only in the livers of the HFD-fed apoCIII mice. The HFD also increased the mRNA levels of SREBP1c (sterol response element binding protein) and one of its targets, ACC (acetyl-CoA carboxylase), independent of genotype. Expression of stearoyl-CoA desaturase-1 (SCD-1) was markedly reduced by the HFD (Figure 3(f)).

Expression levels of genes related to lipid catabolism and secretion are shown in Figure 4. The mRNA level of ATGL (adipose tissue triglyceride lipase), responsible for initial TG hydrolysis, was reduced by HFD consumption independent of genotype. Levels of CPT1 (carnitine palmitoyl acyl transferase) and UCP2 (mitochondrial uncoupling protein-2), which both accelerate fatty acid oxidation, were reduced in the LFD-fed apoCIII mice and in both HFD groups. PGC1*α* (PPAR gamma coactivator-1*α* and inducer of mitochondria biogenesis) levels were reduced by the HFD in both groups. VLDL assembly for subsequent TG secretion is a complex process, involving the association of lipids with apoB, which is mediated by MTP (microsomal triglyceride transfer protein). We observed a reduction in the mRNA abundance of MTP under the HFD in both groups, whereas apoB mRNA was reduced in the apoCIII mice under both diets. In summary, the HFD (independent of genotype) induced the expression of lipogenesis-related genes (SREBP1c and ACC) and decreased the expression of catabolism- and secretion-related genes (SCD1, ATGL, PGC1*α*, and MTP), while apoCIII overexpression (under the LFD) significantly reduced the expression of the CPT1, UCP2 and apoB100 genes.

**Figure 5:**
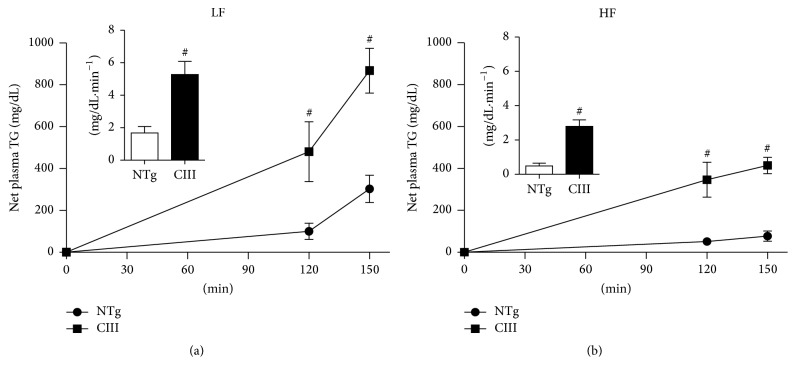
Overexpression of apoCIII increases liver VLDL-TG secretion. Net increases in plasma triglyceride concentrations after intraperitoneal injection of Triton WR 1339 (500 mg/kg) and curve slopes (inserts) for NTg and apoCIII mice fed either a low-fat (LF) or high-fat (HF) diets (*n* = 3–5). Data are expressed as the mean ± SEM. ^#^NTg versus apoCIII mice (*p* < 0.05; Student's *t* test).

To measure the actual liver TG secretion rates under the above experimental conditions, we performed a direct functional assay, as shown in Figure 5. The results show that liver VLDL-TG secretion rates increased in the apoCIII-overexpressing mice regardless of diet and that the HFD reduced VLDL-TG secretion rates in both apoCIII and NTg mice.

### 3.5. Overexpression of ApoCIII Increases the Ratio of Oxidized to Reduced Glutathione in Liver

**Figure 6:**
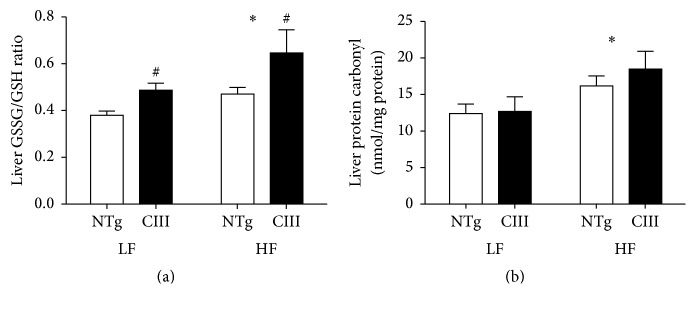
Effects of apoCIII overexpression and high-fat diet consumption on liver oxidative stress and damage. (a) Liver ratio of oxidized to reduced glutathione (*n* = 7–9) and (b) levels of carbonylated proteins (*n* = 6–8) in NTg and apoCIII mice fed either a low-fat (LF) or high-fat (HF) diets for 16 weeks. Data are expressed as the mean ± SEM. ^*∗*^LF versus HF group; ^#^NTg versus apoCIII mice (*p* < 0.05; two-way ANOVA).

Diet-induced NAFLD is associated with cell oxidative stress. Because GSH is one of the most abundant reducing power used to maintain cell redox homeostasis, we measured GSH levels in liver. As shown in Figure 6, overexpression of apoCIII increased the liver GSSG/GSH ratio, independent of diet type, although the HFD exacerbated this effect. Protein carbonylation, which is an indicator of oxidative damage of proteins, was increased by the HFD independent of genotype.

### 3.6. ApoCIII Overexpression Induces Liver Inflammation

**Figure 7:**
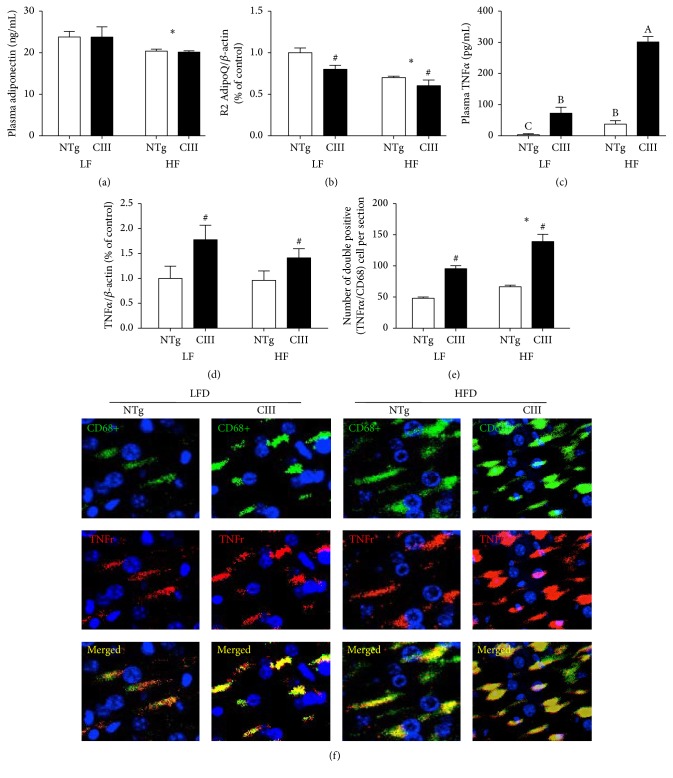
Effects of apoCIII overexpression and high-fat diet consumption on plasma and liver inflammatory markers. (a) Plasma adiponectin (*n* = 7–9), (b) liver adiponectin receptor 2 mRNA (*n* = 7–9), (c) plasma TNF*α* (*n* = 5-6), (d) liver TNF*α* mRNA (*n* = 6–8), (e) quantification of TNF*α* and CD68 colocalized areas (*n* = 3), and (f) representative immunofluorescent confocal microscopy images of TNF*α* and CD68 in liver sections from NTg and apoCIII mice fed either a low-fat (LF) or high-fat (HF) diets. Data are expressed as the mean ± SEM. ^*∗*^LF versus HF group; ^#^NTg versus apoCIII mice (*p* < 0.05; two-way ANOVA). ^A, B, C^Mean values with nonmatching superscript letters are significantly different (*p* < 0.05; ANOVA followed by Bonferroni correction).

Inflammation is a key event in the pathogenesis of NAFLD. Therefore, we measured a panel of systemic and liver proinflammatory markers in the mice. Increased plasma levels of the cytokine IL6 were present in both HFD groups, although greater increases were observed in the apoCIII mice. Another inflammatory marker, C-reactive protein, was significantly increased in the apoCIII mice, independent of diet type (Table 3). Plasma levels of adiponectin, an anti-inflammatory mediator, were diminished by the HFD in both genotypic groups (Figure 7(a)). The levels of liver adiponectin receptor 2 were also reduced by the HFD and in the apoCIII mice, independent of diet type (Figure 7(b)). Therefore, adiponectin signaling appears to be hampered both by HFD consumption and apoCIII overexpression.

The HFD induced elevation of plasma TNF*α* levels, and this effect was more pronounced in the apoCIII mice (Figure 7(c)). Furthermore, liver TNF*α* mRNA levels were increased in the apoCIII mice, independent of diet type (Figure 7(d)). Immunohistochemical analysis confirmed these findings, showing increased expression of the macrophage marker CD68 and colocalization of macrophages with cell-surface TNF*α* receptors in the livers of the apoCIII mice, independent of diet type (Figures 7(e) and 7(f)).

**Figure 8:**
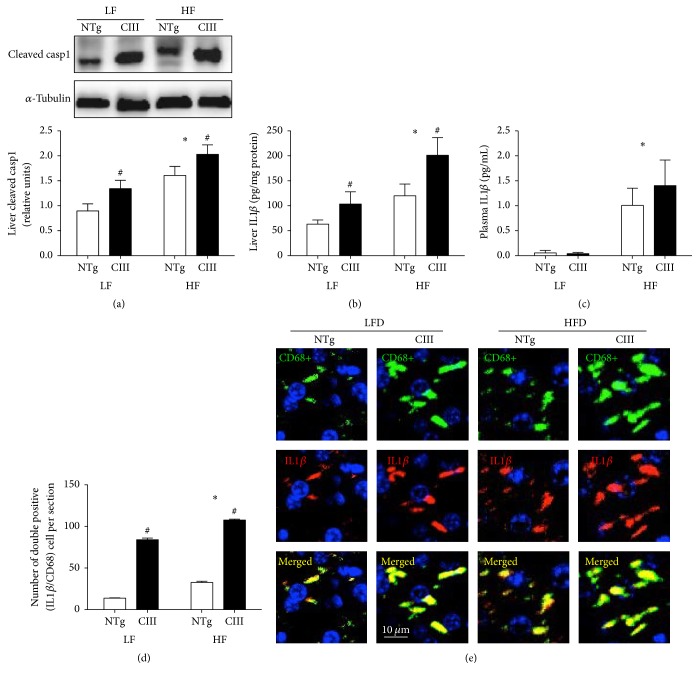
Effects of apoCIII overexpression and high-fat diet consumption on plasma and liver inflammatory markers. (a) Liver cleaved caspase-1 (*n* = 5), (b) liver IL1*ββ* (*n* = 4), (c) plasma IL1*ββ* (*n* = 4-5), (d) quantification of IL1*ββ* and CD68 colocalized areas, and (e) representative immunofluorescent confocal microscopy images of IL1*ββ* and CD68 in liver sections (*n* = 3) from NTg and apoCIII mice fed either a low-fat (LF) or high-fat (HF) diets for 16 weeks. Data are expressed as the mean ± SEM. ^*∗*^LF versus HF groups; ^#^NTg versus apoCIII mice (*p* < 0.05; two-way ANOVA).

The inflammasome pathway appears to be activated in NAFLD. Activation of this pathway involves the formation and activation of a protein multicomplex that contains cysteine-aspartate protease-1 (caspase-1). Activated caspase-1 in turn activates and leads to the subsequent secretion of IL1*β*. Unlike the LFD, the HFD increased the levels of activated (cleaved) caspase-1, although the apoCIII mice exhibited increases in activated caspase-1 levels regardless of diet (Figure 8(a)). The same pattern was observed for IL1*β*: the HFD increased liver IL1*β* levels in both groups, but the apoCIII mice had higher IL1*β* levels independent of diet (Figure 8(b)). In the plasma, we observed that only the HFD affected IL1*β* levels (Figure 8(c)). Immunohistochemical analysis revealed that IL1*β* colocalized with macrophages in the livers of the apoCIII mice, independent of diet type (Figures 8(d) and 8(e)).

### 3.7. ApoCIII Overexpression Induces Liver Apoptosis

**Figure 9:**
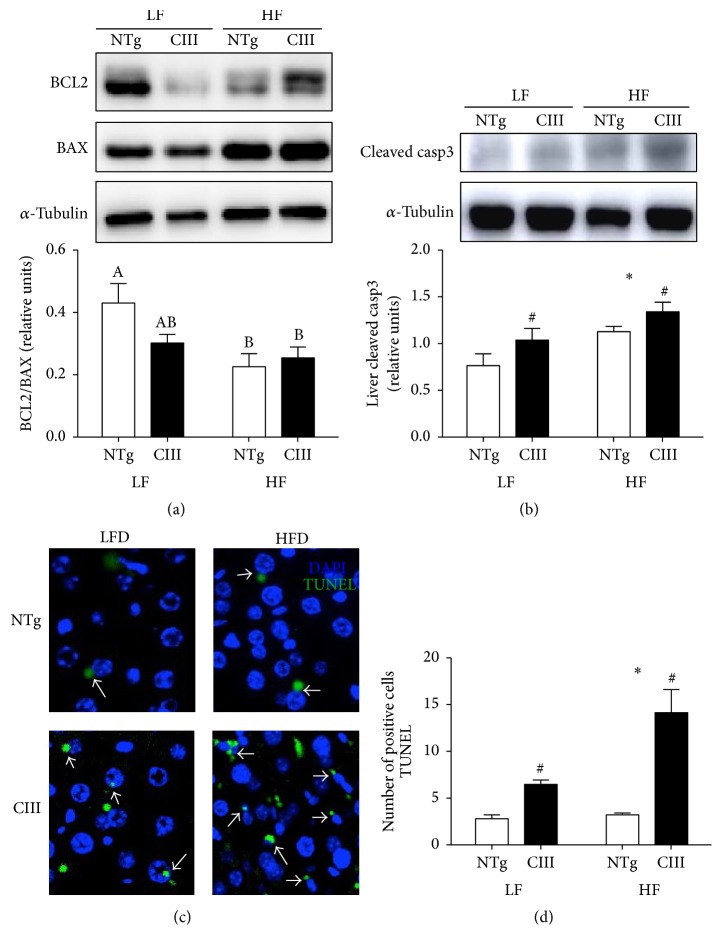
Effects of apoCIII overexpression and high-fat diet consumption on liver cell death. (a) Ratio between the anti- and proapoptotic proteins BCL2 and BAX (*n* = 7–9), (b) cleaved (activated) caspase-3 (*n* = 5-6) and number of apoptotic cells (TUNEL-positive cells), and (c) representative images and (d) quantification (*n* = 3) in NTg and apoCIII mice fed either a low-fat (LF) or high-fat (HF) diets for 16 weeks. Data are expressed as the mean ± SEM. ^*∗*^LF versus HF group; ^#^NTg versus apoCIII mice (*p* < 0.05; two-way ANOVA). ^A, B, AB^Mean values with nonmatching superscript letters are significantly different (*p* < 0.05; ANOVA followed by Bonferroni correction).

Apoptosis is present at a more advanced stage of the disease (steatohepatitis, NASH), which involves both intrinsic and extrinsic cell death pathways. As shown in Figure 9(a), the apoCIII mice exhibited activation of the intrinsic apoptosis signaling pathway, demonstrated by the decreased Bcl2/Bax ratio. The HFD also diminished this ratio in the NTg group. To confirm the occurrence of cell death, we measured the levels of caspase-3, the final death effector, and counted apoptotic cells using a TUNEL assay. Cell death was more prominent in the apoCIII mice (independent of diet) and was further increased by the HFD as evidenced by the high levels of active (cleaved) caspase-3 and high number of TUNEL-positive apoptotic cells (Figures 9(b), 9(c), and 9(d)).

### 3.8. Fibrate Treatment Resolves ApoCIII-Induced Intracellular Lipid Accumulation but Not Inflammation

We next investigated whether NASH could be reversed to the same extent among the different groups of mice. To accomplish this, mice were treated with fenofibrate during the last 2 weeks of HFD consumption. By activating PPAR*α*, this drug modulates the expression of many genes that regulate lipid metabolism, mainly by decreasing synthesis and increasing catabolism. Fibrates are also known to reduce apoCIII expression. As shown in Table 4, fenofibrate treatment reduced body weight, adipose tissue weight, and leptin plasma levels in both HFD groups. Plasma TG, cholesterol, and FFA levels were not affected by fenofibrate treatment and remained elevated in the apoCIII mice. Fenofibrate did not alter PPAR*α* mRNA expression, but it reduced FAS mRNA expression and increased ACO, CPT1, and UCP2 mRNA expression in both HFD groups (Table 5). Fenofibrate also markedly reduced liver TG content. In addition, fenofibrate decreased plasma levels of the hepatic transaminases AST and ALT, although AST levels remained elevated in the apoCIII mice (Table 5).

Fenofibrate treatment was also effective in attenuating both oxidative stress and inflammation (Table 5). GSSG/GSH ratio and carbonyl content were both reduced by fenofibrate, although the latter remained elevated in the apoCIII mice. Additionally, plasma TNF*α* levels were reduced by the drug treatment but remained higher in the apoCIII mice. Plasma adiponectin levels were not altered by fenofibrate, whereas the levels of liver adiponectin receptor 2 were significantly increased only in the NTg mice. Fenofibrate also reduced plasma levels of IL6, but not C-reactive protein (Table 5).

**Figure 10:**
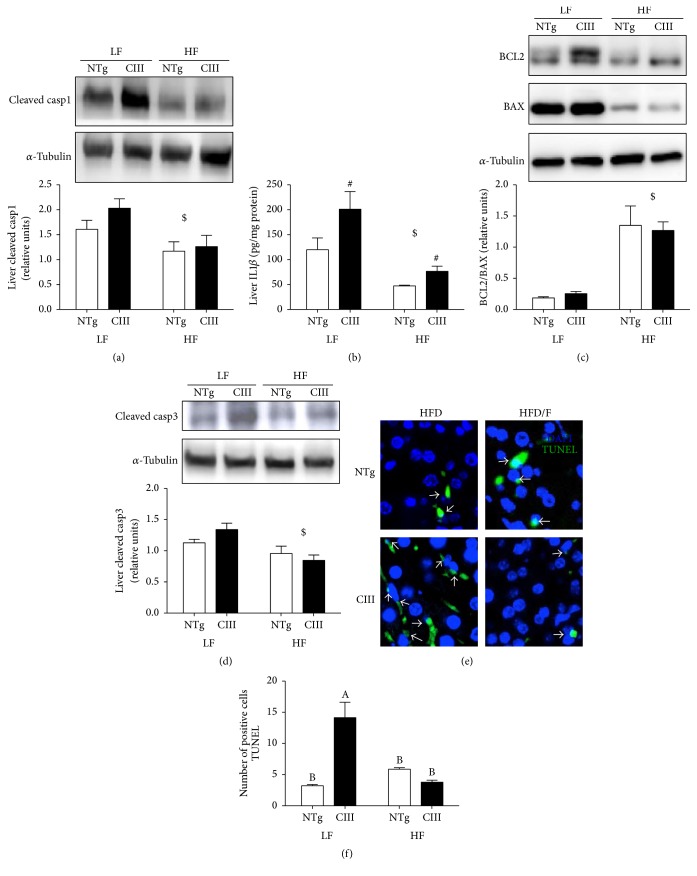
Effects of fenofibrate treatment on high-fat-diet-induced inflammation and apoptosis. (a) Liver cleaved caspase-1 (*n* = 4), (b) liver IL1*ββ* (*n* = 4), (c) liver BCL2/BAX ratio (*n* = 7–9), (d) liver cleaved caspase-3 (*n* = 6) and number of apoptotic cells (TUNEL-positive cells), and (c) representative images and (d) quantification (*n* = 3) from NTg and apoCIII mice fed either a low-fat (LF) or high-fat (HF) diets for 16 weeks. Data are expressed as the mean ± SEM. ^$^LF versus HF group; ^#^NTg versus apoCIII mice (*p* < 0.05; two-way ANOVA). ^A, B^Mean values with nonmatching superscript letters are significantly different (*p* < 0.05; ANOVA followed by Bonferroni correction).

Levels of cleaved caspase-1 (Figure 10(a)) in the liver as well as plasma and liver levels of IL1*β* (Table 5) were diminished by treatment with fenofibrate. However, liver IL1*β* levels (Figure 10(b)) remained elevated in the apoCIII mice independent of treatment. Fenofibrate also increased the Bcl2/Bax ratio (Figure 10(c)) and reduced activated caspase-3 levels in both NTg and apoCIII mice compared to nontreated counterparts. Confirming these results, there was a reduction in the number of TUNEL-positive cells in both fenofibrate-treated groups (Figures 10(d) and 10(e)).

## 4. Discussion

In the present study, we demonstrated that overexpression of apoCIII results in NAFLD, independent of diet. The development of NAFLD was characterized by increases in liver TG content, oxidative stress, inflammation, and cell death. The condition was aggravated and additional NAFLD features were induced, such as impaired glucose tolerance and hepatic insulin resistance, in apoCIII mice consuming a HFD. Furthermore, inflammatory indicators and apoptotic cell numbers were augmented in the HFD-fed apoCIII mice. As expected, fenofibrate treatment reversed several of the effects associated with HFD consumption and apoCIII overexpression. However, it is important to emphasize that fenofibrate did not normalize apoCIII-induced inflammatory traits, such as increased plasma TNF*α* levels and augmented liver TNFr and IL1*β* levels. These findings suggest that persistent hypertriglyceridemia might be more relevant to liver inflammation than intracellular lipid accumulation.

A previous study showed that increased plasma apoCIII concentrations predispose mice to diet-induced NAFLD [21]. These authors focused on the insulin resistance observed in this mouse model when fed a HFD. Severe hepatic insulin resistance was characterized using the hyperinsulinemic-euglycemic clamp technique and was attributed to increased hepatic diacylglycerol content and protein kinase C-epsilon activation and decreased insulin activation of Akt2 [21]. Here, we confirmed that HFD consumption induces insulin resistance more severely in apoCIII mice than in NTg mice, as shown by the simultaneous glucose intolerance and increased gluconeogenesis that developed in the former group. We also confirmed a recent find from our group that apoCIII overexpression aggravates diet-induced obesity [26]. We have previously shown that apoCIII mice exhibit normal glucose homeostasis and insulin secretion when fed a normal diet, and these parameters were disturbed following acute increases in plasma FFA levels induced by heparin [27] or HFD consumption [28].

We showed that apoCIII overexpression increases VLDL-TG secretion regardless of diet type. However, this secretion was not enhanced enough to avoid the accumulation of lipids in the livers of the apoCIII mice. Reductions in the expression of genes related to lipid catabolism (CPT1 and UCP2) were observed in the apoCIII mice, which may explain, at least in part, their higher net liver lipid accumulation. The HFD reduced VLDL-TG secretion rates in both apoCIII and NTg mice, which explains the exacerbations in lipid accumulation that were observed in mice fed this diet. Lee et al. [21] reported increased FFA and TG liver uptake in apoCIII mice regardless of diet type. Others have found that FFA uptake from plasma is increased in liver and skeletal muscle but decreased in adipose tissue in obese subjects with NAFLD compared to obese subjects with normal intrahepatic triglyceride content [29]. Although we found no differences in FAT/CD36 mRNA expression between the apoCIII and NTg mice, it is likely that FFA uptake increased in the apoCIII mice under both diets due to the greater availability of TG and FFA in the plasma of these mice. Interestingly, we found that the HFD induced the expression of ChREBP mRNA specifically in the apoCIII mice. Hepatic de novo lipogenesis is regulated through activation of both SREBP-1c [30] and ChREBP [31], which transcriptionally activate nearly all genes involved in this process. Thus, lipogenesis may also contribute to liver steatosis in apoCIII mice. In addition to increased total TG content, in a previous study, we compared the liver lipidomes of apoCIII and NTg mice fed a regular diet. We demonstrated higher incorporation of oleic acid in phosphatidylcholine and TGs, higher content of phosphatidylinositol-containing arachidonic acid, and distinct overall FFA profiles in the livers of apoCIII mice, which showed elevated relative abundances of oleic (18 : 1), palmitoleic (16 : 1), arachidonic (20 : 4), margaric (17 : 0), and stearic (18 : 0) acids [32]. It is generally accepted that, in the natural course of liver disease, lipid accumulation, either from diet or from de novo lipogenesis, is the triggering event (first hit of the two-hit hypothesis) that leads to lipotoxicity in hepatocytes [16, 33]. In hepatocytes, excessive lipid storage may directly contribute to organelle failure, including mitochondrial dysfunction and endoplasmic reticulum stress and may play a role in hepatic insulin resistance [34]. We previously examined hepatic oxidative status in apoCIII mice fed a regular diet. We found cell redox imbalance, evidenced by increased total levels of carbonylated proteins and malondialdehydes and elevated GSSG/GSH ratios in the livers of apoCIII mice compared to NTg mice [20]. Moreover, we found that the origin of this oxidative stress was associated with higher activities of two oxidases, NADPH oxidase, and xanthine oxidase, whereas mitochondria actually produced lower amounts of H_2_O_2_ due to a mild uncoupling adaptation mediated by the opening of ATP-sensitive potassium channels [20, 35]. Here, we confirmed that the livers of apoCIII mice have lower levels of GSH regardless of diet type, while consumption of a HFD induced protein carbonylation in the livers of both apoCIII and NTg mice.

Recent findings have indicated that inflammation is a key process for both the initiation and progression of NAFLD. For instance, knockout of the caspase-1 gene, which blocks the inflammasome pathway, is sufficient to protect mice from diet-induced NASH [36]. Accordingly, the absence of interleukin 1*α* or 1*β* (both targets of caspase-1) inhibits the evolution of liver steatosis to steatohepatitis and liver fibrosis [37]. In addition, double knockout of TNF*α* receptor 1 and 2 protects mice against liver lipid accumulation and other features of NAFLD induced by consumption of a methionine/choline-deficient diet [38], whereas anti-TNF*α* antibody treatment was shown to decrease lipid content and JNK signaling pathway in HFD-fed ob/ob mice [39]. Huang et al. [40] demonstrated that high-fat or high-sucrose diet-induced steatosis is preventable when the liver is depleted of Kupffer cells, and neutralizing antibodies against TNF*α* attenuate Kupffer cell-induced alterations in hepatocyte lipid metabolism. Our results support the central role of inflammation in NAFLD. In the present study, apoCIII mice fed a LFD exhibited a broad spectrum of liver inflammation, including activation of the TNF*α*, inflammasome, and adiponectin pathways. This inflammation persisted after fibrate treatment, which resolved intracellular lipid accumulation but did not correct the levels of proinflammatory effectors (TNF*α*, TNF*α* receptor, IL1*β*, and low adiponectin-R2).

There are likely multiple origins for the proinflammatory state in this model. It should be noted that none of the inflammatory markers/effectors that were measured in this work are tissue specific. They can be produced locally in the liver by activated Kupffer cells but are also derived from activated circulating immune and vascular cells as well as from adipose tissue, particularly enlarged visceral fat, which is an important source of many cytokines. Mild to moderate hypertriglyceridemia in healthy young men has been associated with increased concentrations of several biochemical markers of inflammation and endothelial activation and dysfunction [41]. This hypertriglyceridemia-associated systemic inflammation can be reversed to varying degrees by administration of fibrates, resveratrol or omega-3 fatty acids [42–44]. However, independent of triglyceride levels, apoCIII has inflammatory and cytotoxic effects [45]. For instance, purified human apoCIII enhances the attachment of monocyte-like cells to the human saphenous vein or coronary artery endothelial cells under both static and laminar shear stress conditions via induction of VCAM-1 [46]. In addition to inflammation, elevated circulating apoCIII levels may contribute to beta-cell apoptosis and diabetes [47–49], although this is controversial [50]. Supporting the finding that apoCIII is proapoptotic, we recently demonstrated that apoCIII-overexpressing spleen mononuclear cells present higher rates of apoptosis in vitro and that apoCIII mice have a reduced number of blood circulating lymphocytes. Additionally, cytochrome c release into cytosol and caspase-8 activity were both increased in apoCIII-overexpressing mononuclear cells, indicating that cell death signaling starts upstream of mitochondria but does involve this organelle [51]. Here, we showed that apoCIII mice exhibit marked increases in apoptotic cell numbers in the liver under low-fat (2-fold) and high-fat (5-fold) diets.

In conclusion, our findings show that, in addition to excessive hepatic lipid accumulation and insulin resistance, apoCIII overexpression-induced hypertriglyceridemia is associated with liver inflammation and cell death, which increase susceptibility to and the severity of diet-induced fatty liver disease.

## Figures and Tables

**Table 1:** Diet composition (g/100 g).

Diet	Protein	Fat	Carbohydrate	Fiber	Calories (Kcal/100 g)
Low fat	14	4 (9.5% of calories)	72	5	380
High fat	14	35 (59% of calories)	41	5	536

*Note*. The basis of both the low-fat and high-fat diets is AIN-93.

**Table 2:** Primer sequences used for RT-PCR.

Genes	Primers	
*β*-Actin	Forward	5′ GGACTCATCGTACTCCTGCTT 3′
	Reverse	5′ GAGATTACTGCTCTGGCTCCT 3′
ACC	Forward	5′ AGGCAGCTGAGGAAGTTGGCT 3′
	Reverse	5′ CGCTGCACAGAGCAGTCACG 3′
ACO	Forward	5′ TGTGACCCTTGGCTCTGTTCT 3′
	Reverse	5′ TGTAGTAAGATTCGTGGACCTCTG 3′
Adiponectin receptor-2	Forward	5′ ACGTTGGAGAGTCATCCCGTA T 3′
	Reverse	5′ CTCTGTGTGGATGCGGAAGAT 3′
Apo B	Forward	5′ GCGAGTGGCCCTGAAGGCTG 3′
	Reverse	5′ CCGTGGAGCTGGCGTTGGAG 3′
ATGL	forward	5′ TGTGGCCTCATTCCTCCTAC C 3′
	Reverse	5′ TCGTGGATGTTGGTGGAGCT 3′
ChREBP	Forward	5′ ACTCAGGGAATACACGCCTACAG 3′
	Reverse	5′ TCTTGGTCTTAGGGTCTTCAGGAA 3′
CPT1	Forward	5′ AGTGACTGGTGGGAGGAATA 3′
	Reverse	5′ CTTGAAGTAACGGCCTCTGT 3′
FAS	Forward	5′ GATATTGTCGCTCTGAGGCTGTTG 3′
	Reverse	5′ GGAATGTTACACCTTGCTCCTTGC 3′
MTP	Forward	5′ CATTCAGCACCTCCGGACTT 3′
	Reverse	5′GATACTGCTGTCACTTTTGAAATCCA 3′
PGC1*α*	Forward	5′ CCTGACACGGAGAGTTAAAGGAA 3′
	Reverse	5′ GATGGCACGCAGCCCTAT 3′
PPAR*α*	Forward	5′ GCAGCTCGTACAGGTCATCA 3′
	Reverse	5′ CTCTTCATCCCCAAGCGTAG 3′
SCD1	Forward	5′ TGGGTTGGCTGCTTGTG 3′
	Reverse	5′ GCGTGGGCAGGATGAAG 3′
SREBP1c	Forward	5′ CCTGGTGGTGGGCACTGAAGC 3′
	Reverse	5′ GCGTCTGAAGGGTGGAGGGGT 3′
TNF*α*	Forward	5′ CCCTCCTGGCCAACGGCATG 3′
	Reverse	5′ TCGGGGCAGCCTTGTCCCTT 3′
UCP2	Forward	5′ AGCATGGTAAGGGACCAGTG 3′
	Reverse	5′ CAGTTCTACACCAAGGCTC 3′

**Table 3:** Food intake; body, liver, and adipose tissue masses; and fasting plasma concentrations of lipids, leptin, and inflammatory markers in NTg and apoCIII mice fed either a low-fat diet (LFD) or a high-fat diet (HFD) for 16 weeks.

	LFD		HFD	
	NTg	CIII	NTg	CIII
Food intake (kcal/mouse/day)	15.41 ± 1.2	14.6 ± 0.9	19.6 ± 0.8^*∗*^	21.8 ± 0.6^*∗*^
	(7)	(7)	(5)	(5)
Body mass (g)	23.2 ± 0.3	24.0 ± 0.3	29.6 ± 1.1^*∗*^	29.5 ± 0.9^*∗*^
	(17)	(16)	(8)	(6)
Liver (%body weight)	3.7 ± 0.0	3.8 ± 0.1	2.9 ± 0.1^*∗*^	3.2 ± 0.1^*∗*^
	(17)	(17)	(8)	(8)
Perigonadal WAT (%)	1.2 ± 0.1	1.4 ± 0.1	3.0 ± 0.2^*∗*^	3.6 ± 0.1^*∗*#^
	(10)	(10)	(8)	(8)
Plasma triglycerides (mg/dL)	71.4 ± 5.0	780.2 ± 53.6^#^	81.5 ± 3.8	668.1 ± 52.8^#^
	(7)	(7)	(7)	(7)
Plasma cholesterol (mg/dL)	122.0 ± 7.8	150.3 ± 11.3	135.1 ± 4.9	199.5 ± 13.2^#^
	(9)	(7)	(7)	(8)
Nonesterified fatty acids (mEq/L)	0.24 ± 0.01	0.50 ± 0.04^#^	0.30 ± 0.03	0.41 ± 0.02^#^
	(10)	(8)	(6)	(5)
Plasma leptin (pg/mL)	635.6 ± 156	526.0 ± 162	1712.8 ± 618^*∗*^	3033.4 ± 925^*∗*^
	(9)	(7)	(6)	(7)
Plasma IL6 (pg/mL)	5.7 ± 0.6	6.5 ± 0.9	13.4 ± 0.9^*∗*^	21.03 ± 2.1^*∗*#^
	(6)	(6)	(7)	(4)
Plasma C-reactive protein (ng/mL)	320.9 ± 31.2	563.1 ± 73.8^#^	405.7 ± 45.8	628.6 ± 39.7^#^
	(9)	(7)	(7)	(8)

Mean ± SEM (*n*). ^*∗*^LFD versus HFD groups; ^#^NTg versus apoCIII mice (*p* < 0.05; two-way ANOVA).

**Table 4:** Food intake; body, liver, and adipose tissue masses; and fasting plasma concentrations of lipids and leptin in NTg and CIII mice fed a high-fat diet (HFD) and treated or not with fenofibrate (HFD/F).

	HFD		HFD/F	
	NTg	CIII	NTg	CIII
Food intake (kcal/animal/day)	19.6 ± 0.8	20.8 ± 0.6	20.1 ± 0.7	20.1 ± 0.8
	(5)	(5)	(4)	(4)
Body weight (g)	28.8 ± 0.5	31.6 ± 2.2	27.4 ± 0.8^$^	28.2 ± 0.3^$^
	(9)	(6)	(6)	(6)
Liver (% body weight)	3.2 ± 0.1	3.0 ± 0.1^#^	3.6 ± 0.1^$^	3.4 ± 0.1^#$^
	(7)	(8)	(6)	(6)
Perigonadal WAT (%)	3.0 ± 0.3	3.5 ± 0.3	1.8 ± 0.2^$^	2.1 ± 0.4^$^
	(6)	(7)	(6)	(6)
Plasma triglycerides (mg/dL)	83.2 ± 4.9	693.0 ± 28.3^#^	59.0 ± 4.7	508 ± 59.6^#^
	(7)	(7)	(7)	(7)
Plasma cholesterol (mg/dL)	132.9 ± 5.2	187.2 ± 14.2^#^	154.3 ± 11.6	232.7 ± 30.6^#^
	(6)	(6)	(6)	(6)
Nonesterified fatty acids (mEq/L)	0.33 ± 0.03	0.40 ± 0.01^#^	0.25 ± 0.02	0.48 ± 0.05^#^
	(7)	(8)	(6)	(6)
Plasma leptin (pg/mL)	1783.4 ± 143	2011.5 ± 266	644.69 ± 57^$^	704.1 ± 52^$^
	(6)	(5)	(6)	(6)

Mean ± SEM (*n*). ^$^HFD versus HFD/F groups; ^#^NTg versus apoCIII mice (*p* < 0.05; two-way ANOVA).

**Table 5:** Plasma indicators of liver injury and inflammation, liver triglyceride content, oxidative stress indicators, and gene expression related to energy metabolism in NTg and CIII mice fed a high-fat diet (HFD) with or without fenofibrate treatment (HFD/F).

	HFD		HFD/F	
	NTg	CIII	NTg	CIII
Plasma AST (U/L)	123.3 ± 9.8	160.6 ± 7.7^#^	74.5 ± 13.7^$^	98.6 ± 11.8^#$^
	(5)	(5)	(5)	(5)
Plasma ALT (U/L)	77.3 ± 11.4	107.7 ± 12.5^#^	35.7 ± 5.8^$^	39.9 ± 10.9^#$^
	(5)	(5)	(4)	(4)
Plasma adiponectin (ng/mL)	20.2 ± 0.6	20.13 ± 0.3	19.86 ± 0.5	19.12 ± 0.2
	(5)	(5)	(5)	(6)
Plasma TNF*α* (pg/mL)	37,51 ± 11.3	301.05 ± 17.5^#^	13.3 ± 7.0^$^	140.1 ± 7.0^#$^
	(4)	(4)	(4)	(4)
Plasma IL6 (pg/mL)	13.64 ± 1.3^b^	26.52 ± 2.1^a^	14.2 ± 1.8^b^	14.38 ± 1.4^b^
	(5)	(5)	(5)	(5)
Plasma C-reactive protein (ng/mL)	415.2 ± 44.1	582.6 ± 32.5	586.8 ± 22.1	530.9 ± 29.0
	(5)	(5)	(5)	(5)
Plasma IL1*β* (pg/mL)	1.00 ± 0.34	1.40 ± 0.51	0.53 ± 0.08	0.54 ± 0.07
	(4)	(4)	(4)	(5)
Liver triglycerides (mg/g)	11.36 ± 1.1	14.0 ± 2.6	7.10 ± 0.8^$^	8.66 ± 1.5^$^
	(7)	(8)	(6)	(6)
Liver GSSG/GSH ratio	0.53 ± 0.01	0.54 ± 0.02	0.49 ± 0.01^$^	0.47 ± 0.04^$^
	(6)	(6)	(6)	(6)
Liver protein carbonyl (nmol/mg protein)	15.1 ± 1.5	17.2 ± 2.4^#^	7.1 ± 0.5^$^	12.2 ± 2.0^#$^
	(6)	(7)	(6)	(6)
Liver mRNA (% control)				
PPAR*α*	1.0 ± 0.1	1.0 ± 0.1	1.1 ± 0.2	1.1 ± 0.2
	(5)	(6)	(5)	(6)
FAS	1.0 ± 0.2	1.2 ± 0.1	0.39 ± 0.1^$^	0.45 ± 0.1^$^
	(8)	(5)	(5)	(5)
CPT1	1.0 ± 0.1	0.62 ± 0.1	1.2 ± 0.2^$^	1.1 ± 0.2^$^
	(8)	(8)	(6)	(6)
ACO	1.0 ± 0.3	1.1 ± 0.1	1.7 ± 0.1^$^	1.4 ± 0.2^$^
	(6)	(6)	(6)	(6)
UCP2	1.0 ± 0.1	0.9 ± 0.1	2.0 ± 0.1^$^	2.0 ± 0.3^$^
	(6)	(5)	(5)	(5)
Adiponectin R2	1.0 ± 0.1^b^	0.54 ± 0.05^c^	1.70 ± 0.2^a^	0.56 ± 0.1^c^
	(8)	(6)	(6)	(6)

mRNA data normalized to beta-actin. Mean ± SEM (*n*) ^$^HFD versus HFD/F groups and ^#^NTg versus apoCIII mice (*p* < 0.05; two-way ANOVA). ^a, b, c^Mean values with nonmatching superscript letters are significantly different (*p* < 0.05, ANOVA followed by Bonferroni correction).

## Abbreviations

ACC: Acetyl-CoA carboxylase
ACO: Acetyl-CoA oxidase
AdipoQr2: Adiponectin receptor 2
ALT: Alanine aminotransferase
Apo B: Apolipoprotein B
Apo CIII: Apolipoprotein CIII
AST: Aspartate aminotransferase
ATGL: Adipose tissue triglyceride lipase
Bax: Bcl2-associated X protein
Bcl2: B-cell lymphoma 2
CD68: Cluster of differentiation 68
ChREBP: Carbohydrate response element binding protein
CPT1: Carnitine palmitoyl acyl transferase 1
CVD: Cardiovascular disease
DAPI: Diamidino-2-phenylindole
FAS: Fatty acid synthase
FFA: Free fatty acid
GSH: Reduced glutathione
GSSG: Oxidized glutathione
HFD: High-fat diet
IL-1*β*: Interleukin-1 beta
IL-6: Interleukin-6
ITT: Insulin tolerance test
LFD: Low-fat diet
MTP: Microsomal triglyceride transfer protein
NADPH: Nicotinamide adenine dinucleotide phosphate
NAFLD: Nonalcoholic fatty liver disease
NASH: Nonalcoholic steatohepatitis
NTg: Nontransgenic
OGTT: Oral glucose tolerance test
PBS: Phosphate-buffered saline
PGC1*α*: PPAR gamma coactivator 1 alpha
PPAR*α*: Peroxisome proliferator-activated receptor alpha
RT-PCR: Real-time polymerase chain reaction
SCD1: Stearoyl-CoA desaturase-1
SREBP1c: Sterol response element binding protein
TG: Triglycerides
TNFr1: Tumor necrosis factor receptor 1
TNF*α*: Tumor necrosis factor
TUNEL: Deoxynucleotidyl-mediated deoxyuridine triphosphate end labeling
UCP2: Mitochondrial uncoupling protein-2
VCAM-1: Vascular cell adhesion molecule 1
VLDL: Very low-density lipoprotein.
